# Epidemiology and risk factors for invasive fungal infections during induction chemotherapy for newly diagnosed acute myeloid leukemia: A retrospective cohort study

**DOI:** 10.1371/journal.pone.0197851

**Published:** 2018-06-08

**Authors:** Ming-Yu Lien, Chia-Hui Chou, Ching-Chan Lin, Li-Yuan Bai, Chang-Fang Chiu, Su-Peng Yeh, Mao-Wang Ho

**Affiliations:** 1 Division of Hematology and Oncology, Department of Internal Medicine, China Medical University Hospital, Taichung City, Taiwan, R.O.C; 2 Graduate Institute of Basic Medical Science, China Medical University, Taichung City, Taiwan, R.O.C; 3 Division of Infection Disease, Department of Internal Medicine, China Medical University Hospital, Taichung City, Taiwan, R.O.C; 4 Clinic of Hematology and Oncology, Department of Internal Medicine, Graduate Institute of Clinical Medicine, China Medical University, Taichung City, Taiwan, R.O.C; Cardiff University, UNITED KINGDOM

## Abstract

This study investigated the epidemiology and risk factors associated with invasive fungal infections (IFIs) during induction chemotherapy in a cohort of Taiwanese patients with newly-diagnosed acute myeloid leukemia (AML). IFIs are a significant complication in the management of immunocompromised cancer patients; such infections are associated with a high incidence of morbidity and mortality, particularly in many South-Asian countries, where IFI rates are increasing. We retrospectively analyzed IFI incidence data from 105 patients with newly diagnosed AML at a single center undergoing their first course of induction chemotherapy without primary antifungal prophylaxis between November 2008 and December 2014. Of 21 cases documented as proven/provable IFIs 16 (76%) were invasive aspergillosis, 2 (10%) were mucormycosis infections, and 3 (14%) were proven yeast infections. The lung was the most commonly affected site (n = 16; 76%); 2 patients (10%) developed fungal sinusitis. IFI cases were more often males (*P* = 0.020). In multivariate analysis, patients with neutropenia lasting>30 days were more than twice as likely to develop IFI (OR, 2.24 [95% CI, 2.81–31.11], *P*<0.001). We also confirmed patients with smoker and receiving parenteral nutrition during chemotherapy were significant associated with IFIs. Our findings suggest that antifungal prophylaxis should be considered for patients with AML during induction chemotherapy, particularly in patients from Southeastern Asia, an area of potentially high IFI rates. We recommend that clinicians determine which patients receiving induction chemotherapy for AML are at high risk of developing IFI, to allow for targeted therapeutic prophylaxis.

## Introduction

Despite recent advances in the field of hematologic malignancies, such as non-invasive biomarkers for early diagnosis, radiographic screening and the widespread use of novel antifungal drugs[1, 2], invasive fungal infections (IFIs) remain a major cause of morbidity and mortality in patients with acute myeloid leukemia (AML)[3, 4]. Scant data exist as to IFI epidemiology in Asia, especially in the subtropical or tropical regions, where the environments are particularly suitable for fostering fungal growth. In 2015, a 2-year observational study determined that the epidemiology of invasive fungal diseases among patients with hematologic disorders in 8 Asian countries/regions was similar to that in Western centers, with an overall 30-day mortality of 22.1%[5]. However, the patient population did not consist of high-risk individuals. Another recent report has indicated a high incidence of IFI in AML patients receiving induction chemotherapy in Southeastern Asia[6]. However, this study did not identify the risk for IFI. Stratifying patients at risk for IFI could potentially determine those who would be most likely to benefit from more intensive antifungal treatment.

The present study sought to describe the epidemiology, clinical characteristics, risk factors and outcomes of IFI in patients with newly diagnosed AML receiving their first induction treatment in Taiwan, in a setting that did not routinely use primary antifungal prophylaxis.

## Design and methods

This retrospective cohort study was conducted in the hematology wards of China Medical University Hospital, Taiwan, between November 2008 and December 2014. Enrollment was limited to adult patients (aged over 18 years) with newly diagnosed AML undergoing their first induction chemotherapy. We reviewed patients’ records from the time of hospital registration and up until 100 days after completion of chemotherapy. All study procedures complied with the ethical standards of the Research Ethic Committee of China Medical University Hospital, Taiwan, and the basic ethical principles contained in the Helsinki Declaration for studies in humans. The requirement of informed consent was waived for this study by the Research Ethic Committee because the data were analyzed anonymously (Ethics committee approval record: CMUH101-IRB1-144). All patients gave written consent before enrollment.

Baseline data were recorded for each patient at admission and included age, sex, cigarette smoking history, AML subtype, cytogenetic risk, and existing comorbidities (e.g., COPD, diabetes). Additional hospitalization data indicated whether patients were kept in positive pressure isolation rooms with air filtered by high-efficiency particulate air (HEPA) units, as well as individual risk factors (e.g., use of a central venous catheter, parenteral nutrition, duration of neutropenia). The diagnostic workup included nasal, pharyngeal, and rectal swabs at the time of admission, blood cultures and chest radiography at onset of fever, weekly galactomannan (GM) assays, and chest computed tomography (CT) imaging at 7 days after onset of fever. Additional examinations (e.g., abdominal ultrasound scan, sinus or brain CT, skin biopsy, bronchoalveolar lavage, or fundus examination) were performed as required.

AML treatment regimens were similar for approximately 80% of the patient cohort throughout the entire study period. Standard induction therapy typically consisted of idarubicin (12 mg/m^2^) on days 1–3 and cytarabine (100–200 mg/m^2^ daily) on days 1–7. The remaining 20% of patients were administered non-standard regimens for AML subtype M3, or they were older-aged patients (>60 years): patients with acute promyelocytic leukemia (AML subtype M3) received idarubicin combined with retinoic acid; older patients received a reduced-dose anthracycline-based regimen, e.g., I2A5 (idarubicin for 2 days combined with cytarabine for 5 days), or daunorubicin combined with cytarabine. IFI incidence was assessed within the first 100 days after completing chemotherapy. Invasive fungal diseases were classified according to the 2008 criteria published by the EORTC Mycoses Study Group (MSG)[7].

For the assessment of risk factors for IFI, patients with possible mold infections were assigned to the control group for analysis. Patients without any evidence of proven/probable mold infection or proven yeast infection served as controls and were compared with IFI cases. None of the patients received bacterial or antifungal prophylaxis. Patients who had febrile neutropenia were treated with a broad-spectrum beta-lactam, with or without an aminoglycoside, as per guidelines issued by the Infectious Diseases Society of America[8]. Empirical antifungal treatment was initiated in cases of persistent or recurrent fever unresponsive to broad-spectrum antibiotics.

### Statistical analysis

Values are expressed as the mean± standard deviation or median (range) of a continuous variable, or as a percentage of the group from which they were derived (categorical variable). Categorical variables were compared using the chi-square test between IFI cases (proven/probable) and negative control groups. Subsequently, a backward stepwise multi-variates logistic regression was used to identify those factors independently associated with the onset the invasive fungal infection. Adjusted Odd ratio (OR) and 95% confidence intervals(CI) were calculated. We did another sensitivity analysis to examine the impact of “possible IFIs” in the control group. The statistical analysis was performed using SPSS software for Windows software (version 17.0).

## Results

### Patient characteristics

We analyzed data from induction remission courses in 105 patients with newly diagnosed AML 94 of whom were diagnosed with de novo AML and 7 with promyelocytic leukemia. As shown in Table 1, the median age of patients was 51 years (range, 19 to 76 years), with a median body mass index of 23 kg/m^2^ (range, 14 to 30 kg/mg^2^) and 67 (64%) of the participants were male. Approximately one-third (n = 34; 32%) of the cohort were current smokers. All study participants received a standard-dose, anthracycline-based remission-induction regimen. All patients received each treatment session in the hospital; 34 (32%) were admitted to HEPA-filter rooms. At diagnosis, 17 (16%) patients were in the favorable-cytogenetic group, 72 (69%) in the intermediate-risk group and 16 (15%) in the unfavorable group according to the Medical Research Council classification. Following induction therapy, 59 patients (56%) achieved a clinical remission (CR) and 20 (19%) achieved a partial remission (PR). Response to induction chemotherapy was undefined in three patients; these patients had no data from a bone marrow examination after chemotherapy.

**Table 1:** Baseline characteristics of 105 Taiwanese patients with newly diagnosed acute myeloid leukemia (AML).

Characteristics	Value (%)
Demographics	
Males	67 (64)
Median age in years (range)	51 (19–76)
Cigarette smoking	34 (32)
Diabetes	7 (7)
Median body mass index (kg/m^2^) (range)	23 (14–30)
Admitted to a HEPA-filter room (%)	34 (32)
AML classification	
De novo AML	94 (90)
M3	7 (7)
MDS-related changes*	11 (10)
Cytogenetic risk	
Favorable	17 (16)
Intermediate	72 (69)
Unfavorable	16 (15)
First remission-induction chemotherapy	
Anthracycline-based regimen	100 (100)
Response to induction chemotherapy	
Complete remission	59 (56)
Partial remission	20 (19)
Resistant disease	23 (22)
Central venous catheter	87 (83)
Parenteral nutrition	29 (28)
Median duration of neutropenia (days) (range)	30 (10–78)
Overall mortality (within 100 days)	9 (9)
IFI-attributable mortality (within 100 days)	6 (6)

* MDS-related changes were independent of de novo AML and M3 status.

Response to induction chemotherapy was undefined in 3 patients; these patients had no data from a bone marrow examination after chemotherapy.

HEPA = high-efficiency particulate air; MDS = myelodysplastic syndrome; IFI = invasive fungal infection.

During the treatment period, central venous catheters were implanted in 87 patients (83%). Twenty-nine (28%) patients received parenteral nutrition at admission. The median duration of neutropenia was 33 days (Table 1). Of all 105 courses of induction chemotherapy, 35 (33%) all-category IFIs were identified, including 7 (7%) proven, 14 (13%) probable and 14 (13%) possible IFIs. Of the 21 etiological pathogens identified in the proven or probable IFIs, 18 (86%) were mold, including 14 that were exclusively diagnosed using positive GM test results. Two patients with mucormycosis were diagnosed by culture histopathology. The majority of identified yeast infections were bloodstream infections. *Candida tropicalis* was the most common yeast identified (2 cases). The lung was the most commonly affected site (n = 16; 76%). Two (10%) patients had fungal sinusitis and 3 (15%) patients had disseminated IFI (candidemia) (Table 2).

**Table 2:** Clinical characteristics of 21 cases of proven or probable invasive fungal infection among 105 Taiwanese patients with new diagnosed acute myeloid leukemia.

Clinical characteristics	Value (%)
Site of infection	
Lung	16 (76)
Sinuses	2 (10)
Disseminated	3 (14)
Certainty of diagnosis	
Proven	7 (33)
Probable	14 (66)
Fungal species	
Yeast	3 (14)
*Candida* spp.	1
*Candida tropicalis*	2
Mold	18 (86)
Positive galactomannan tests only	14
*Aspergillus* spp.	2
Mucormycosis	2

### Risk factors for IFI

Twenty-one patients with proven/probable IFIs were compared with 84 controls (Table 3). Patients with IFI were more often male (86% vs 58%; *P* = 0.020). There were no significant between-group differences for age, type of AML (primary vs secondary), or cytogenetic risk category. No significant associations were found between IFI and cigarette smoking (*P* = 0.056), diabetes (*P* = 0.118), BMI (P = 0.171), WBC (*P* = 0.296), platelet levels (*P* = 0.486), or post-induction status. An analysis of post-treatment variables showed an increased risk of IFI in patients with prolonged neutropenia (i.e., >30 days; *P*<0.001) and in those with prolonged thrombocytopenia (i.e., ≥30 days; *P*<0.001). Receipt of parenteral nutrition was associated with IFI (27% of controls vs 52% of cases; *P* = 0.029). Admission to isolation rooms with HEPA filters and insertion of central venous catheters did not appear to represent additional risk factors.

**Table 3:** Univariate analysis of the risk factors for proven/probable invasive fungal infection (IFI) in 105 Taiwanese patients with newly diagnosed acute myeloid leukemia (AML).

Variables	Controls (N = 84)		IFI cases (N = 21)		*P*-value
	n	%	n	%	
Age, year					0.741
<60	61	73	16	76	
≥60	23	27	5	24	
Sex					0.020*
Female	35	42	3	14	
Male	49	58	18	86	
Type of AML					0.152
Primary	77	92	17	81	
Secondary	7	8	4	19	
Cytogenetic risk category					0.272
Favorable	69	82	15	71	
Unfavorable	15	18	6	29	
Smoking status					0.056*
No	62	74	11	52	
Yes	22	26	10	48	
Diabetes mellitus					
No	80	95	18	86	0.118
Yes	4	5	3	14	
Body mass index (kg/m^2^)					
<18	7	8	0	0	0.171
≥18	77	92	21	100	
Isolation room					0.144
No	54	64	17	81	
Yes	30	36	4	19	
Catheterization					0.612
0	16	19	3	14	
1	68	81	18	86	
Parenteral nutrition					0.029*
No	61	73	10	48	
Yes	23	27	11	52	
WBC					0.296
<10,000	34	40	11	52	
≥10,000	50	60	10	48	
Platelet					0.486
<15,000	18	21	6	29	
≥15,000	66	79	15	71	
Neutropenia days					<0.001*
0–30	59	70	5	24	
>30	25	30	16	76	
Thrombocytopenia days					<0.001*
<30	46	55	2	10	
≥30	38	45	19	90	
Post-induction status					0.169
CR	50	60	9	43	
Non-CR	34	40	12	57	

* In Chi-square testing, a P-value of < 0.05 was considered to be statistically significant.

Significant variables in univariate analysis were included and adjusted in the multivariate analysis with Cox regression, for evaluating the risk of proven/probable IFIs. Prolonged thrombocytopenia was not included, due to its highly statistical association with prolonged neutropenia. As shown in Table 4, multivariate analysis confirmed that onset of proven/probable IFIs was significantly influenced by three variables: prolonged neutropenia (>30 days) (OR, 2.24; 95% CI, 2.81–31.11; *P*<0.001), receipt of parenteral nutrition (OR, 1.17; 95% CI, 1.05–9.90; *P* = 0.041) and smoking status (OR, 1.16; 95% CI, 1.01–10.08; P = 0.048). No such association was observed for sex and IFI in multivariate analysis.”

**Table 4:** Multivariate analysis of the risk factors for proven/probable invasive fungal infection during induction chemotherapy among Taiwanese patients with newly diagnosed acute myeloid leukemia.

Variables	Proven/Probable Fungal Infection		
	OR	95% CI	*P*-value
Neutropenia >30 days	2.24	2.81–31.11	<0.001*
Parenteral nutrition	1.17	1.05–9.90	0.041*
Smoking	1.16	1.01–10.08	0.048*

* A P-value of < 0.05 was considered to be statistically significant. OR = odds ratio; CI = confidence interval.

In sensitivity analysis, significant positive correlation was observed between IFI and four variables, including males(P = 0.013), non-unfavorable cytogenetic risk(P = 0.018), smoking (P = 0.04), parenteral nutrition((P = 0.009), and prolonged neutropenia days (P<0.001). The multivariate analysis still confirmed that onset of proven/probable IFIs was significantly influenced by the prolonged neutropenia (lasting >30 days) (OR, 1.85 [95% CI, 1.77–22.88], P = 0.005) and receipt of parenteral nutrition (OR, 1.63 [95% CI, 1.42–18.52], P = 0.013).

## Discussion

This retrospective cohort study confirmed a high incidence of IFI in AML patients undergoing induction chemotherapy. We found that smoking status, parenteral nutrition and neutropenia lasting >30 days were significant independent variables associated with IFI in this patient cohort. Patients with hematologic malignancies have been historically categorized as being at high risk for IFI(9); the incidence of IFI is highest amongst patients with AML during the neutropenic period after induction chemotherapy. We therefore focused our analysis on a cohort of hematologic patients with newly diagnosed AML who received remission-induction chemotherapy with a standard regimen. In order to decrease the heterogeneity of the study population; we excluded those patients who received lower-intensity chemotherapy regimens. Our study included 7 patients with acute promyelocytic leukemia, all of whom were treated with the same anthracycline-based standard-dose regimen.

The incidence rate of proven or probable IFI was 20% during the 6-year study period. When combined with possible cases, the IFI rate increased to 33%. Compared with other studies, the incidence of IFI in our report exceeds rates reported in Western countries[9–12]. It is recognized that IFI rates vary between studies due to different study designs, underlying diseases and treatment periods. Our study result was similar to that from another recent report from Taiwan, a Southeast Asian country with local climactic conditions (humid and warm) that favor the growth of fungal infections(6). However, we did not identify any association between rainy season months and IFI development. Italian researchers reported a strong association between pre-hospitalization exposure to sources of fungi and the development of IFI occurring within 30 days after the first course of chemotherapy in adult patients with newly diagnosed AML(14). In that study, a significant association was found between invasive mold infections and environmental factors including job, hobbies, and recent house renovations. For all countries/regions, local epidemiological information for IFI is extremely important, because infection control practices in AML patients differ between hospitals[13]. In our clinical practice, we did not use antifungal prophylaxis in AML patients receiving induction chemotherapy, which may explain our high IFI incidence.

The inhalation of fungal spores plays an important role in IFI development[14]. In our study, pulmonary fungal infections were identified early, because our patients were closely monitored by weekly GM antigen testing as well as lung CT imaging performed on day 7 of fever. In our patients, 76% of IFIs were primarily localized in the lung, followed by blood stream infection (14%) and sinus (10%). In 2011, researchers from a single Taiwanese hospital reported that, over a 15-year period, invasive fungal sinusitis (IFS) was more common in AML patients with prolonged neutropenia[15]. In that investigation, the overall 6-week mortality rate was 41% amongst IFS cases compared with 13% amongst non-IFS case. Serum *Aspergillus* GM antigen was elevated in 7 of 11 patients (64%) with IFS caused by aspergillosis but negative for all 3 patients with mucormycosis. In our study, GM antigen levels were elevated in 1 of 2 patients with proven sinus aspergillosis. This patient remained alive after aggressive surgical debridement combined with antifungal therapy. Thus, early IFI diagnosis through serial GM testing could enable the early introduction of antifungal therapy and surgery, and potentially lower mortality in AML patients receiving induction chemotherapy.

In 2005, a review of changing patterns in risk factors for systemic fungal infections reported that invasive aspergillosis was the predominant IFI in patients with hematologic malignancies, following documented increased use of voriconazole and echinocandin[16]. Since then, there has been a slight increase in mucormycosis frequency, due to the limited activity of these antifungals against *Mucorales* species[17]. In our study, we identified 2 patients with mucormycosis, 1 of whom died. In our study, most fungemia cases were caused by infection with non-albicans *Candida* species. Two of 3 cases infected with *C*. *tropicalis* were successfully treated with fluconazole and survived.

A previous prospective study revealed patients with newly diagnosed AML to be at higher risk if they were smokers only in the univariate analysis (19). In recent study, BT Hill found that higher IFI incidence was developed in patients received allogeneic hematopoietic stem cell transplantation(allo-HCT) with heavy-smoking than non-smokers. (27.5% vs 15.9%). And the increased rate of complications from fungal infections in heavy smokers may lead to a decrease in overall survival (20). Aspergillus spores are found in tobacco cigarettes, burning contaminated tobacco leads to spore release, thereby further increasing the exposure of immunocompromised patients (21). In addition, due to immune cell damage and failure to activate intracellular signaling, smoking has been shown to have suppressive effect on the immune protective system of alveolar macrophages, dendritic cells, natural killer (NK) cells and neutrophils (22). In our study, there was a significantly association between smoking status and invasive fungal infections on multivariable analysis.

The common side effects of induction chemotherapy for newly diagnosed AML patients include nausea, vomiting and severe mucositis, which can worsen nutrition status. We used parenteral nutrition for these patients. Few studies have evaluated the nutritional status in patients with AML undergoing first induction chemotherapy. In recent research, 18% of an AML cohort had low nutritional status, accompanied by low body mass index (BMI) values and weight loss after induction chemotherapy(23). In that study, BMI values were significantly higher in patients with normal nutritional status (27.7 kg/m^2^) compared with their counterparts with low nutritional status (20.1 kg/m^2^; *P*<0.0001). The median BMI in our study was 23 kg/m^2^ (14–30 kg/m^2^). Due to low BMI values, almost a third (28%) of our patients used parenteral nutrition; receipt of parenteral nutrition during the treatment period was a significant variable associated with IFI in our AML cohort. This finding is in agreement with previous research, which has determined that total parenteral nutrition increases the risk for candidemia[18–22]. The high prevalence of candidemia may reflect the long-term use of intravascular catheters[23]. However, we found no such correlation between central venous catheters and IFI in our data. A continuous, high-dextrose concentration infusion has frequently been associated with hyperglycemia. In one study, a cohort of allogeneic hematopoietic cell transplant patients who developed hyperglycemia were at increased risk for invasive fusariosis[24]. Similarly, other researchers have demonstrated that acute leukemia patients with hyperglycemia were more likely to develop complicated infections (including IFI)[25]. In a previous study that examined the association between parenteral nutrition and IFI, most participants were critically ill ICU patients who did not have acute leukemia. To the best of our knowledge, parenteral nutrition has not been previously investigated in patients with invasive aspergillosis, nor in a prospective study involving a large sample of patients. In our analysis, we found a significant correlation between parenteral nutrition and mold IFI, as confirmed by multivariate analysis. This higher-risk group of patients should be strongly considered as being suitable candidates for early administration of antifungal therapy.

We also confirmed an association between the duration of neutropenia (lasting >30 days) and risk of IFI. Neutrophils play an important role in the control of fungal infection. With an increased proliferation of leukemia cells in the bone marrow, production of normal neutrophils declines[26]. Lack of neutrophil recovery usually indicates refractory acute leukemia. Brazilian researchers have developed an index (the D-index) that combines duration and severity of neutropenia and successfully predicts different risks for invasive mold infection in febrile neutropenic patients (33). This index was not available for us to apply at the start of induction therapy. Moreover, baseline neutrophil counts could not predict the onset of IFI in our analysis.

This study had several limitations, including its retrospective setting, the small number of patients, and it was conducted in a single tertiary care institution. We were unable to obtain details of parenteral nutrition formulation or daily parenteral caloric intake. The decision to commence enteral nutrition was based on the attending physician’s decision, determined by different clinical conditions. Thus, the length of time required for intestinal nutrition differed for individual patients. Furthermore, we did not record antihyperglycemic agent or insulin dose administered to each patient. Finally, our risk factor analysis did not include patients with possible mold infections, which might have resulted in an underestimation of the true infection incidence. Nevertheless, despite these limitations, our study still has significance for clinical practice and future research.

In conclusion, we confirmed a high incidence of IFI cases in our institution in Taiwan. Antifungal prophylaxis should be considered in patients with AML during their first course of induction chemotherapy. Pre-chemotherapy smoking status, prolonged neutropenia and parenteral nutrition administered during chemotherapy were associated with IFI in our AML cohort. We recommend that close monitoring of fungal infections and early use of antifungal drugs may reduce the risk of IFI during parenteral nutrition. Future prospective research should be undertaken to determine the effect of antifungal prophylaxis given during remission-induction therapy for leukemia.

## References

[1] Ruping MJ, Vehreschild JJ, Cornely OA. Patients at high risk of invasive fungal infections: when and how to treat. Drugs. 2008;68(14):1941–62. . 18778118 10.2165/00003495-200868140-00002

[2] Kontoyiannis DP. Invasive mycoses: strategies for effective management. The American journal of medicine. 2012;125(1 Suppl):S25–38. doi: 10.1016/j.amjmed.2011.10.009 . 22196206 10.1016/j.amjmed.2011.10.009

[3] Pagano L, Caira M, Candoni A, Offidani M, Martino B, Specchia G, et al Invasive aspergillosis in patients with acute myeloid leukemia: a SEIFEM-2008 registry study. Haematologica. 2010;95(4):644–50. doi: 10.3324/haematol.2009.012054 . 19850903 10.3324/haematol.2009.012054 PMC2857195

[4] Michallet M, Benet T, Sobh M, Kraghel S, El Hamri M, Cannas G, et al Invasive aspergillosis: an important risk factor on the short- and long-term survival of acute myeloid leukemia (AML) patients. European journal of clinical microbiology & infectious diseases: official publication of the European Society of Clinical Microbiology. 2012;31(6):991–7. doi: 10.1007/s10096-011-1397-5 . 21909650 10.1007/s10096-011-1397-5

[5] Hsu LY, Lee DG, Yeh SP, Bhurani D, Khanh BQ, Low CY, et al Epidemiology of invasive fungal diseases among patients with haematological disorders in the Asia-Pacific: a prospective observational study. Clinical microbiology and infection: the official publication of the European Society of Clinical Microbiology and Infectious Diseases. 2015;21(6):594 e7–11. doi: 10.1016/j.cmi.2015.02.019 . 25749561 10.1016/j.cmi.2015.02.019

[6] Tang JL, Kung HC, Lei WC, Yao M, Wu UI, Hsu SC, et al High Incidences of Invasive Fungal Infections in Acute Myeloid Leukemia Patients Receiving Induction Chemotherapy without Systemic Antifungal Prophylaxis: A Prospective Observational Study in Taiwan. PLoS One. 2015;10(6):e0128410 doi: 10.1371/journal.pone.0128410 . 26061179 10.1371/journal.pone.0128410 PMC4462587

[7] De Pauw B, Walsh TJ, Donnelly JP, Stevens DA, Edwards JE, Calandra T, et al Revised definitions of invasive fungal disease from the European Organization for Research and Treatment of Cancer/Invasive Fungal Infections Cooperative Group and the National Institute of Allergy and Infectious Diseases Mycoses Study Group (EORTC/MSG) Consensus Group. Clinical infectious diseases: an official publication of the Infectious Diseases Society of America. 2008;46(12):1813–21. doi: 10.1086/588660 . 18462102 10.1086/588660 PMC2671227

[8] Freifeld AG, Bow EJ, Sepkowitz KA, Boeckh MJ, Ito JI, Mullen CA, et al Clinical practice guideline for the use of antimicrobial agents in neutropenic patients with cancer: 2010 update by the infectious diseases society of america. Clinical infectious diseases: an official publication of the Infectious Diseases Society of America. 2011;52(4):e56–93. doi: 10.1093/cid/cir073 . 21258094 10.1093/cid/cir073

[9] Ananda-Rajah MR, Grigg A, Downey MT, Bajel A, Spelman T, Cheng A, et al Comparative clinical effectiveness of prophylactic voriconazole/posaconazole to fluconazole/itraconazole in patients with acute myeloid leukemia/myelodysplastic syndrome undergoing cytotoxic chemotherapy over a 12-year period. Haematologica. 2012;97(3):459–63. doi: 10.3324/haematol.2011.051995 . 22058198 10.3324/haematol.2011.051995 PMC3291603

[10] Malagola M, Peli A, Damiani D, Candoni A, Tiribelli M, Martinelli G, et al Incidence of bacterial and fungal infections in newly diagnosed acute myeloid leukaemia patients younger than 65 yr treated with induction regimens including fludarabine: retrospective analysis of 224 cases. European journal of haematology. 2008;81(5):354–63. doi: 10.1111/j.1600-0609.2008.01122.x . 18637030 10.1111/j.1600-0609.2008.01122.x

[11] Neofytos D, Lu K, Hatfield-Seung A, Blackford A, Marr KA, Treadway S, et al Epidemiology, outcomes, and risk factors of invasive fungal infections in adult patients with acute myelogenous leukemia after induction chemotherapy. Diagnostic microbiology and infectious disease. 2013;75(2):144–9. doi: 10.1016/j.diagmicrobio.2012.10.001 . 23142166 10.1016/j.diagmicrobio.2012.10.001 PMC3986043

[12] Hammond SP, Marty FM, Bryar JM, DeAngelo DJ, Baden LR. Invasive fungal disease in patients treated for newly diagnosed acute leukemia. American journal of hematology. 2010;85(9):695–9. doi: 10.1002/ajh.21776 . 20652970 10.1002/ajh.21776

[13] Perfect JR, Hachem R, Wingard JR. Update on epidemiology of and preventive strategies for invasive fungal infections in cancer patients. Clinical infectious diseases: an official publication of the Infectious Diseases Society of America. 2014;59 Suppl 5:S352–5. doi: 10.1093/cid/ciu639 . 25352630 10.1093/cid/ciu639

[14] Haleem Khan AA, Mohan Karuppayil S. Fungal pollution of indoor environments and its management. Saudi journal of biological sciences. 2012;19(4):405–26. doi: 10.1016/j.sjbs.2012.06.002 . 23961203 10.1016/j.sjbs.2012.06.002 PMC3730554

[15] Chen CY, Sheng WH, Cheng A, Chen YC, Tsay W, Tang JL, et al Invasive fungal sinusitis in patients with hematological malignancy: 15 years experience in a single university hospital in Taiwan. BMC infectious diseases. 2011;11:250 doi: 10.1186/1471-2334-11-250 . 21939544 10.1186/1471-2334-11-250 PMC3196720

[16] Richardson MD. Changing patterns and trends in systemic fungal infections. The Journal of antimicrobial chemotherapy. 2005;56 Suppl 1:i5–i11. doi: 10.1093/jac/dki218 . 16120635 10.1093/jac/dki218

[17] Lewis RE, Cahyame-Zuniga L, Leventakos K, Chamilos G, Ben-Ami R, Tamboli P, et al Epidemiology and sites of involvement of invasive fungal infections in patients with haematological malignancies: a 20-year autopsy study. Mycoses. 2013;56(6):638–45. doi: 10.1111/myc.12081 . 23551865 10.1111/myc.12081

[18] Gaspar GG, Menegueti MG, Auxiliadora-Martins M, Basile-Filho A, Martinez R. Evaluation of the predictive indices for candidemia in an adult intensive care unit. Revista da Sociedade Brasileira de Medicina Tropical. 2015;48(1):77–82. doi: 10.1590/0037-8682-0292-2014 . 25860468 10.1590/0037-8682-0292-2014

[19] Leon C, Ruiz-Santana S, Saavedra P, Galvan B, Blanco A, Castro C, et al Usefulness of the "Candida score" for discriminating between Candida colonization and invasive candidiasis in non-neutropenic critically ill patients: a prospective multicenter study. Critical care medicine. 2009;37(5):1624–33. doi: 10.1097/CCM.0b013e31819daa14 . 19325481 10.1097/CCM.0b013e31819daa14

[20] Ostrosky-Zeichner L, Sable C, Sobel J, Alexander BD, Donowitz G, Kan V, et al Multicenter retrospective development and validation of a clinical prediction rule for nosocomial invasive candidiasis in the intensive care setting. European journal of clinical microbiology & infectious diseases: official publication of the European Society of Clinical Microbiology. 2007;26(4):271–6. doi: 10.1007/s10096-007-0270-z . 17333081 10.1007/s10096-007-0270-z

[21] Stratman RC, Martin CA, Rapp RP, Berger R, Magnuson B. Candidemia incidence in recipients of parenteral nutrition. Nutrition in clinical practice: official publication of the American Society for Parenteral and Enteral Nutrition. 2010;25(3):282–9. doi: 10.1177/0884533610368704 . 20581323 10.1177/0884533610368704

[22] Luzzati R, Cavinato S, Giangreco M, Grana G, Centonze S, Deiana ML, et al Peripheral and total parenteral nutrition as the strongest risk factors for nosocomial candidemia in elderly patients: a matched case-control study. Mycoses. 2013;56(6):664–71. doi: 10.1111/myc.12090 . 23675641 10.1111/myc.12090

[23] Nielsen XC, Chen M, Hellesoe AM, Jeppesen PB, Gyldenlykke J, Tvede M, et al Etiology and epidemiology of catheter related bloodstream infections in patients receiving home parenteral nutrition in a gastromedical center at a tertiary hospital in denmark. The open microbiology journal. 2012;6:98–101. doi: 10.2174/1874285801206010098 . 23248717 10.2174/1874285801206010098 PMC3520033

[24] Garnica M, da Cunha MO, Portugal R, Maiolino A, Colombo AL, Nucci M. Risk factors for invasive fusariosis in patients with acute myeloid leukemia and in hematopoietic cell transplant recipients. Clinical infectious diseases: an official publication of the Infectious Diseases Society of America. 2015;60(6):875–80. doi: 10.1093/cid/ciu947 . 25425628 10.1093/cid/ciu947

[25] Buyukcelik A, Yalcin B, Utkan G, Doruk H. Relation between the duration of remission and hyperglycemia during induction chemotherapy for acute lymphoblastic leukemia with a hyperfractionated cyclophosphamide, vincristine, doxorubicin, and dexamethasone/methotrexate-cytarabine regimen. Cancer. 2004;101(5):1100–1; author reply 1. doi: 10.1002/cncr.20461 . 15329922 10.1002/cncr.20461

[26] Pagano L, Akova M, Dimopoulos G, Herbrecht R, Drgona L, Blijlevens N. Risk assessment and prognostic factors for mould-related diseases in immunocompromised patients. The Journal of antimicrobial chemotherapy. 2011;66 Suppl 1:i5–14. doi: 10.1093/jac/dkq437 . 21177404 10.1093/jac/dkq437

